# Effect of ropivacaine on peripheral neuropathy in streptozocin diabetes-induced rats through TRPV1-CGRP pathway

**DOI:** 10.1042/BSR20190817

**Published:** 2019-11-13

**Authors:** Nanwen Zhang, Haixiang Wei, Weifang Wu, Peimin Lin, Yuan Chen, Zhiwei Liu, Honglin Wang, Yize Bian, Kai Yu, Shan Lin, Yanqi Cui, Renwei Luo, Jianming Lin, Xiaole Chen

**Affiliations:** 1 Department of Pharmacology, The School of Pharmacy, Fujian Medical University, Fuzhou, P.R. China; 2 Fujian Provincial Key Laboratory of Natural Medicine Pharmacology, Fuzhou, P.R. China; 3 Department of Anesthesiology, The First Hospital of Nanping, Fujian Medical University, Nanping, P.R. China; 4 Department of Anesthesiology, Fuzhou Children’s Hospital of Fujian, Fuzhou, P.R. China; 5 The School of Clinical Medicine, Fujian Medical University, Fuzhou, P.R. China

**Keywords:** calcitonin gene-related peptide, diabetes, peripheral nerve, rat, ropivacaine

## Abstract

**Objective** To determine the effect of ropivacaine on peripheral neuropathy in diabetic rats and its possible mechanism.

**Methods** Forty-eight Sprague–Dawley rats were randomly divided into six groups: nondiabetic control group, nondiabetic group A (0.25% ropivacaine), nondiabetic group B (0.75% ropivacaine), diabetic control group (diabetic peripheral neuropathy (DPN) +artificial cerebrospinal fluid), diabetic group A (DPN+0.25% ropivacaine), and diabetic group B (DPN + 0.75% ropivacaine), with eight rats in each group. Within an hour of the last administration, the sciatic motor nerve conduction velocity (MNCV) of each group was measured, and the morphological changes of rat sciatic nerve were observed by HE, Weil’s staining and electron microscopy. The expression of transient receptor potential vanilloid (TRPV1) in the spinal cord dorsal horn of rats was analyzed by immunohistochemistry, and the expression of Calcitonin gene-related peptide (CGRP) protein in the spinal cord was analyzed by Western blot.

**Results** Compared with the nondiabetic control group, elevated blood glucose, decreased weight and reduced average mechanical withdrawal threshold (MWT), additionally, the sciatic nerves showed significantly slowed conduction velocity (both *P*<0.001) and damaged pathological structure, the expression of TRPV1 and CGRP were decreased (both *P*<0.001) in the diabetic groups. Compared with the diabetic control group, down-regulation of TRPV1 and CGRP in spinal cord was significant for the diabetic groups A and B treated with 0.25 and 0.75% ropivacaine, the higher concentration of ropivacaine correlated with a greater change.

**Conclusion** Ropivacaine can significantly block sciatic nerve conduction velocity in DPN rats in a concentration-dependent manner, which may be related to the expression of the TRPV1-CGRP pathway.

## Introduction

Diabetic peripheral neuropathy (DPN) is the most common complication of type 1 and 2 diabetes. DPN is defined as the presence of symptoms and/or signs of peripheral nerve dysfunction in diabetic patients after the exclusion of other causes [1]. Anesthetic management of these patients is more challenging, with more frequent difficulties in airway control, association with myocardial dysfunction and renal disease, and the occurrence of perioperative dysglycemia. For upper or lower limb surgery in diabetic patients, peripheral regional anesthesia is a potential alternative to general anesthesia because it provides effective analgesia, may decrease hemodynamic complications, and can reduce glycemia dysregulation. Diabetic patients with neuropathy may have increased risk because of the possibility for double crush syndrome, when a chronic axon lesion related to diabetes is associated with an unexpected distal nerve injury related to regional anesthesia [2].

Neuropeptides are recently described neurotransmitters with extensive biological activities. Of the studied neuropeptides, the Calcitonin gene-related peptide (CGRP) has received much attention, yet only few studies have explored the role of CGRP on peripheral neuropathy and its mechanism. CGRP has strong biological activity, acting as an important neurotransmitter in both the central and peripheral nervous systems (CNS and PNS) [3,4]. By coupling G protein to transmit signals into cells through cAMP, CGRP is key to many physiological effects, making it an active participant in the development and progression of DM, pain, inflammation, and other diseases or disease-related symptoms.

The release of CGRP depends on the presence of the transient receptor potential vanilloid (TRPV) on the sensory nerve endings or membranes of neurons. TRPV1 is also an important nociceptor related to neurogenic inflammation. A study by Hong et al. [5] showed more oxidative stress in Streptozotocin (STZ)-induced DM rats (6–8 weeks) in spinal cord dorsal root ganglion and greater cellular damage compared with rats in the control group. These damages were reduced in capsaicin-treated rats through activity related to the activation of TRPV1, indicating a role of TRPV1 activity in the early stage of DM.

Ropivacaine is a commonly used clinical anesthetic with low cardiovascular and central nervous system toxicity and accurate anesthetic and analgesic effects [6]. It is widely used in local infiltration, postoperative analgesia, nerve block anesthesia and analgesia, adult epidural block and analgesia, subarachnoid block and postoperative sacral analgesia in children, and as an epidural analgesia during childbirth [7–10]. Zhi et al. [11] tested application of 0.375 and 0.5% ropivacaine on sciatic nerve electrophysiology with DM rats, including motor nerve conduction velocity (MNCV) of sciatic nerve, the action potential wave amplitude, and the latent periods of 15 min and 48 h after use. Ropivacaine at both concentrations increased damage of sciatic nerve to DM rats, with more serious damage in the 0.5% group.

**Figure 1:**
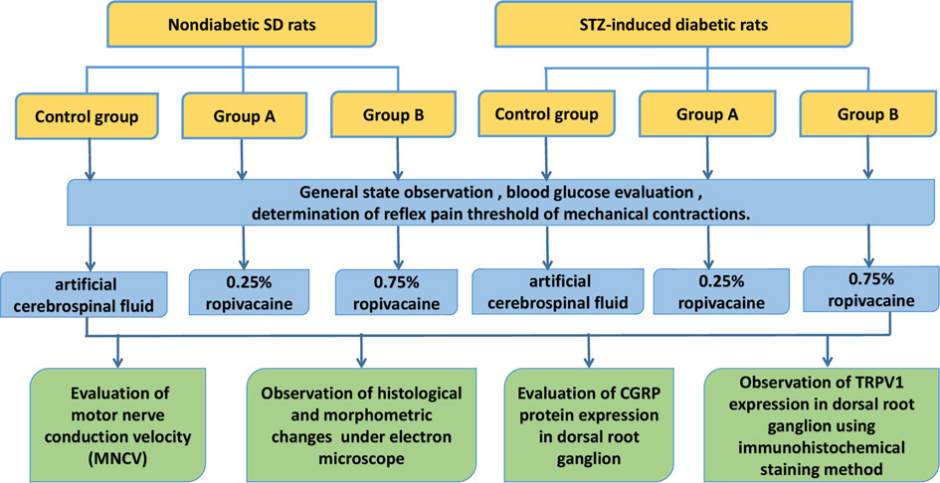
Experimental scheme to determine the effect of intrathecally injected ropivacaine at common dosages on nerve conduction velocity and CGRP expression in the spinal dorsal horn in STZ-induced DPN rats

Overall, diabetes is a worldwide health problem that seriously threatens human health. Long-term diabetes can lead to atrophy of the dorsal root ganglion (DRG), thus causing the degradation of the peptidergic primary sensory nervous system. The resulting variation of plasma CGRP level may affect the progression of diabetes. Patients with diabetes, particularly long-term patients, have higher risks during the perioperative period. Because ropivacaine is a commonly used local anesthesia, it is important to determine if ropivacaine treatment can delay nervous conduction in patients with diabetes, especially DPN patients. Further, it is necessary to determine if this complication is related to the expression of CGRP in the spinal cord. The objectives of the present study were to examine the effect of intrathecally injected ropivacaine at common dosage on nerve conduction velocity and determine the expression of CGRP in the spinal cord of STZ-induced DPN rats. The insights provided by the present study should help assess the risks of ropivacaine use for DPN patients to guide appropriate anesthetic strategy (Figure 1).

## Materials and methods

### Animals

Forty-eight sterile, male Sprague–Dawley (SD) rats, weighing 250 ± 50 g, were provided by the Experimental Animal Center of Fujian Medical University (Certificate No. SCXK (Fujian) 2016-0002), where the animal work has taken place. The rats were housed in groups of three in plastic shoebox cages with sawdust bedding in a room maintained at a temperature of 22 ± 2°C, with a 12–12 h light/dark cycle and humidity maintained between 45 and 55%. Cages were cleaned every 1–2 days. Animals had free access to food and water, and received a normal diet. Animals were habituated to the person performing the experiment and to the testing equipment. After 1 week, animals were randomly and blindly assigned to different treatment groups. All procedures were performed in accordance with protocols approved by the Ethics Review Committee for Animal Experimentation of Fujian Medical University (No. 2018-049). All animal handling procedures were performed in strict accordance with the care of laboratory animals according to the Fujian Province Zoological Society.

### Establishment of DPN rat model

Before the establishment of models, food was withheld for 12 h, but rats received free access to water. On the day of model establishment, STZ (Sigma Chemical Co., St. Louis, MO) was dissolved in 4°C, 0.1 mmol.L^−1^ citric acid buffer (pH = 4.5) and was made at 2% concentration, filtered to sterilize, and then used immediately. Before injection, each rat was weighed and then a one-shot injection of 2% STZ (60 mg/kg) was administered to the left lower abdomen. All surgical steps were performed in 15 min to ensure the sterility of the whole process. After 72 h, blood samples were removed from the tail tip and blood glucose levels were measured using a Roche glucometer. A fasting blood glucose (FBG) level ≥ 16.7 mmol/l was used as the standard to evaluate whether the rats developed diabetes. We selected 24 successfully modeled rats for the experiment, and placed them as three rats per cage. Another 24 healthy rats constituted the nondiabetic group, and these rats were injected with the same volume of sodium citrate buffer.

### Blood glucose evaluation

Venous blood glucose levels were measured in the second, the fourth and the eighth week after DM modeling. Using a fixation-machine, we exposed the tail and applied 75% ethyl alcohol. The top 2–3 mm of the tail was cut, allowing the flow of venous blood. The blood was applied to the appropriate position on the test paper and the value was displayed on the recorder.

### Determination of the reflex pain threshold of mechanical contractions

In order to assess the successful construction of the DPN model, we used an electronic Von Frey liquid crystal display automatic pain gauge to detect the mechanical withdraw threshold (MWT) of the mechanical shrinkage of rats [12]. We evaluated the change between the pre-model base measurement and measurements made 2, 4 and 8 weeks after DM model establishment.

Three days before each experiment, we put rats on the experimental table for 30 min to allow adjustment to the environment. When performing an experiment, we put rats in a Perspex hood on a metal grid mat, and allowed the rats to adapt for 10 min before the experiment. During this period, the rats showed high activity, often accompanied by defecation. Any excrement on the grid was cleaned carefully. After 10 min of adjustment, we used a set of 15 nylon ropes of different radius. The bending strength of the nylon ropes varied between 0.1 and 65 kg. We selected a 0.7-mm vonFrey fiber to stimulate the middle area of the lower right foot of the rat, and then added pressure gradually until the rats showed an explicit response (like licking the feet, suddenly contracting or shaking the feet). When a response was observed, we recorded the reading on the machine, which represented the mechanical constricting reflex threshold of the rat. Each rat was tested three times, 5 min apart and the outcomes were averaged.

### Drug administration

Fifty-six days (8 weeks) after DPN model establishment, the nondiabetic rats were randomly separated into three experimental groups:

1. In the nondiabetic control group, healthy rats were injected with 50 μl artificial cerebrospinal fluid;
2. In nondiabetic group A, healthy rats were injected with 50 μl of 0.25% ropivacaine;
3. In nondiabetic group B, healthy rats were injected with 50 μl of 0.75% ropivacaine;
4. Similarly, the diabetic rats were randomly separated into three experimental groups;
5. In the diabetic control group, DPN-modeled rats were injected with 50 μl of artificial cerebrospinal fluid;
6. In diabetic group A, DPN-modeled rats were injected with 50 μl of 0.25% ropivacaine;
7. In diabetic group B, DPN-modeled rats were injected with 50 μl of 0.75% ropivacaine. The dosages tested were selected based on published references [13–15] and the outcome of a preliminary experiment. To apply the dosage, intrathecal injection was performed daily for 7 days. An hour after the final injection, samples were collected and measured.

### Evaluating motor nerve conduction velocity

After withholding food for 12 h, we used Isoflurane (RWD Co., Shenzhen, China) for anesthesia and then fixed the rats upside down. We removed the fur in the surgical area, and then cut the skin with a surgical blade approximately 0.5 cm below the femur. The cut was made parallel to the femur, exposing the muscle. We then lifted the muscle and cut it, revealing the white sciatic nerve, which extends from the sciatic notch, down to the ankles. The nerve was slightly separated to avoid destroying it. After separation, the nerve was maintained at 37°C and the test was carried out immediately. Saline was used as a conductive medium. A stimulation needle electrode was placed at the outgoing part of the sciatic nerve, between the femoral tuberosity and the ischial tuberosity, indicating the position at which the sciatic nerve divides into the tibial nerve and the peroneal nerve. Two stimulation needles were placed at the point where the tibial and fibula muscles were fully separated. A ground needle electrode was placed in the shallow gluteus muscle and connected to a 6240B/C multichannel physiological recorder. We selected the experimental part of the nerve trunk to determine the MNCV and used the machine as a synchronous trigger [16], recording the current waveform, without stacking; a positive voltage stimulus at a stimulation frequency of 500 Hz, with single twitch stimulation; followed by a 5.0 ms delay. The wave width was 0.1 ms, at an intensity of 1.0 V, and this was then repeated. The latency of the action potential of the distal sciatic nerve was recorded, and the distance between the two recording electrodes was measured. MNCV was calculated as the distance between the two recording electrodes divided by the difference in spike latency. We repeated the electrical stimulation three times and averaged the results.

### Histological and morphometric studies

For pathologic studies, sciatic nerve blocks were obtained from the proximal region of the thigh to the proximal knee joint to its point of division into the common peroneal, tibial and sural nerves. After removal, the sciatic nerve tissues were paraffin-embedded and sectioned. Tissue samples 5-μm-thick were stained with Hematoxylin and Eosin and Weil’s stain [17], and then analyzed (×400; Leica microscope). For morphometric studies, three sections from each animal were randomly chosen. Nerve cells were analyzed for degenerative change, vacuolization and/or demyelination, according to previously described histopathological evidence for STZ-induced neuropathy.

### Observation of sciatic nerves by transmission electron microscopy

The sciatic nerves were pre-fixed with 2.5% glutaraldehyde solution and post-fixed with a 1% osmium tetroxide solution, followed by gradient dehydration with ethanol and acetone. The samples were then saturated, embedded, polymerized and solidified with pure epoxy resin Epon 812 (SPI CHEM, U.S.A.). Ultra-thin sections of the sciatic nerves were prepared, stained with uranyl acetate for 30 min and washed three times with distilled water. The moisture was removed by filter paper and then by natural drying. The samples were then stained with lead citrate for 30 min and washed three times with distilled water. The moisture was again absorbed with filter paper and then allowed to dry naturally [18].

### Determination of TRPV1 expression in spinal cord dorsal horn by immunohistochemical staining

The spinal cord tissues were paraffin-embedded and sectioned. The sections were dewaxed using conventional methods [18], then heated for 10 min at 92–98°C in citrate solution (pH 6.0). Next, the samples were cooled in 3% H_2_O_2_ solution for 10 min at room temperature, 5% bovine serum albumin (BSA) was added and the sections were incubated at room temperature for 20 min. The excess fluid was poured out, and 150 μl (1:100 dilution) of the primary antibody (rabbit anti-rat TRPV1 polyclonal antibody) was added, and allowed to react at 37°C for 60 min. For the negative control group, 0.1 mol.l^−1^ PBS was added in place of the primary antibody. Two-step immunoassay reagents were added to the sections, followed by incubation at 37°C for 30 min. Next, 150 μl of freshly prepared DAB solution (MXB Biotechnology, Fuzhou, China) was added, and the samples were observed under the microscope. After washing with distilled water, the sections were stained with Hematoxylin. After conventional dehydration and vitrification, the sections were cover-slipped with neutral resin and observed. The results were considered positive if the tissues were stained brown and the nuclei were stained blue. A Leica microscope, Leica image acquisition system, and Image-Pro Plus 6.0 image analysis software were used to analyze the immunohistochemical results and measure the integral optical density (IOD) values of each visual field.

### Determination of CGRP expression in spinal cord by Western blot

The lumbar spinal cord tissue was dissected by laminectomy and samples were immediately placed in liquid nitrogen, then stored at −80°C. Protein extracts from lumbar spinal cord tissues were prepared in RIPA buffer (50 mM Tris/HCl, pH 8.0, 150 mM NaCl, 1.0% NP-40, 0.5% sodium deoxycholate and 0.1% SDS) containing protease and phosphatase inhibitors for 30 min at 4°C. Thirty microgram samples of lysate were resolved by SDS/PAGE, transferred to PVDF membrane, and then probed with CGRP antibody and horseradish peroxidase (HRP)-conjugated secondary antibodies (CST, U.S.A.). β-Actin was used as a loading control, and proteins were detected using an ECL chemiluminescence detection kit (Yeasen Biotech Co., Ltd. Shanghai, China) [19].

### Statistical analysis

All results are presented as mean values ± standard error of the mean (SEM). The statistical analysis was performed by one-way analysis of variance (ANOVA) using GraphPad Prism 8.0. For all the tests, differences of *P*<0.05 were considered significant.

## Results

### The change of weight during the modeling process

**Figure 2:**
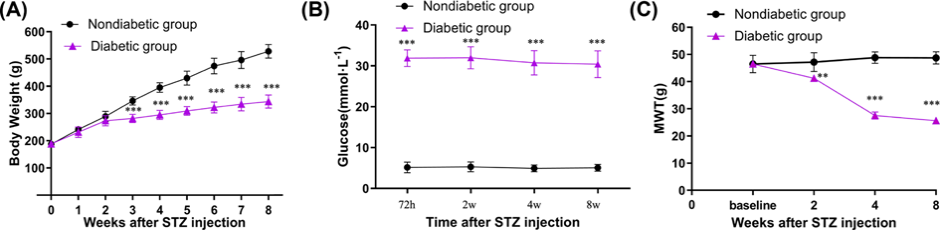
Changes in diabetic group rats (**A**) The change of weight during the modeling process. (**B**) The change of blood glucose during the modeling process. (**C**) The change of reflex pain threshold during the modeling process. Values are expressed as mean ± S.E.M. (*n*=24 in each group). Data were analyzed by one-way ANOVA followed by Tukey’s multiple test. Data were compared with data from the control group over the same period. **P<0.01, ***P<0.001.

During the modeling process, with increased time, the rats in the nondiabetic and diabetic groups gained significant weight, with less of an increase in the weight of the rats in the diabetic groups. The differences in weight gain between rats in the nondiabetic and diabetic groups were significant (*P*<0.01) by the third week, and the differences remained at 5 weeks (Figure 2A).

### The change of blood glucose during the modeling process

The blood glucose levels in the caudal vein were significantly increased relative to the levels in the diabetic groups, 72 h (3 days) after STZ injection (*P*<0.01). As the modeling proceeded, the rats in the diabetic group exhibited a high level of blood glucose on 14 days (2 weeks), 28 days (4 weeks), and 56 days (8 weeks) compared with the levels in the nondiabetic groups measured at the same time (*P*<0.01) (Figure 2B).

### The change of the mechanical withdrawal threshold during the modeling process

The mechanical withdrawal threshold values for the nondiabetic and diabetic rats were (46.48 ± 3.23) and (46.49 ± 1.13) g. Two weeks after being injected with STZ, the average mechanical withdrawal threshold of the hind foot of diabetic rats decreased to (41.21 ± 0.97) g, which was significantly different from the value for the nondiabetic group rats (*P*<0.01). In the fourth week, the average mechanical withdrawal threshold of the diabetic rats decreased further, to (27.53 ± 1.31) g, again significantly different from that of the nondiabetic group rats (*P*<0.001). The threshold decreased slightly from the fourth to the eighth week, to (25.64 ± 1.20) g, still significantly different from the nondiabetic rats (*P*<0.001). The average mechanical withdrawal threshold of the diabetic rats in the eighth week was decreased by 44.8% compared with the threshold value before modeling, which demonstrated that the DPN model was successfully established (Figure 2C).

### Effect of ropivacaine on the conduction velocity of sciatic nerve in rats

After 8 weeks of modeling with STZ, the experimental results showed that the mechanical withdrawal threshold of diabetic groups, compared with nondiabetic groups, were significantly decreased, indicating that DPN model was built successfully. Thereafter, according to the experimental plan, rats were treated with ropivacaine or artificial cerebrospinal fluid once a day for 7 days. To observe the effect of different concentrations of ropivacaine on the conduction velocity of sciatic nerve in DPN rats and its possible mechanism.

The MNCV of the sciatic nerve was used as the reference index to determine the effect of ropivacaine on sciatic neuropathy in diabetic rats. As shown in Table 1, compared with the nondiabetic control group, the MNCV values of nondiabetic groups A and B decreased by 12.92 and 23.55%, respectively, while the diabetic control group, diabetes group A, and diabetes group B decreased by 40.02, 44.08 and 83.44% (both *P*<0.001). Compared with the MNCV value of the diabetic control group, there was no significant change in the sciatic nerve conduction velocity in diabetic group A, but the conduction velocity of diabetic group B was significantly changed, 72.39% (*P*<0.001).

**Table 1:** Effect of ropivacaine on the conduction velocity of sciatic nerve in rats (*n*=8)

Groups	MNCV (m/s)	Inhibition ratio 1 (%)	Inhibition ratio 2 (%)
Nondiabetic control group (artificial cerebrospinal fluid 50 μl)	65.70 ± 2.83	-	-
Nondiabetic group A (ropivacaine 0.25% 50 μl)	57.21 ± 3.61^1^	12.92	-
Nondiabetic group B (ropivacaine 0.75% 50 μl)	50.94 ± 7.08^1^	23.55	-
Diabetic control group (artificial cerebrospinal fluid 50 μl)	39.41 ± 4.11^1^	40.02	-
Diabetic group A (ropivacaine 0.25% 50 μl)	36.74 ± 3.79^1^	44.08	2.67
Diabetic group B (ropivacaine 0.75% 50 μl)	10.88 ± 4.00^1,2^	83.44	72.39

The inhibition rate 1 represents the degree of decline in MNCV values compared with the nondiabetic control group. The inhibition rate 2 means the degree of decline in MNCV values compared with the diabetic control group. Values are expressed as mean values ± S.E.M. Data were analyzed by using one-way ANOVA followed by Tukey’s multiple test. Compared with the nondiabetic control group, ^1^*P*<0.01. Compared with the diabetic control group, ^2^*P*<0.01.

### Effect of ropivacaine on the pathomorphological changes of the sciatic nerve in DPN rats by HE staining

**Figure 3:**
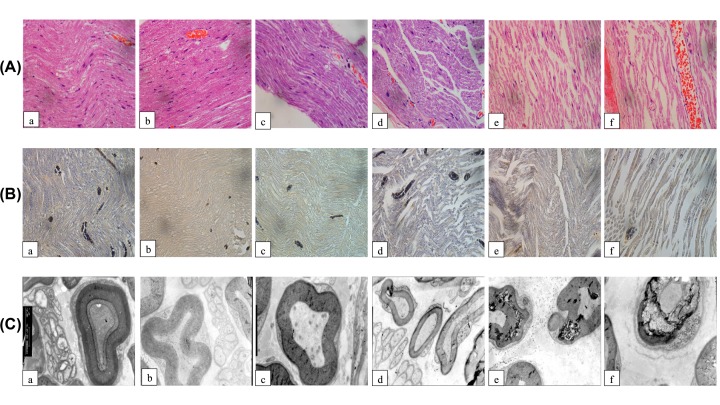
Effect of ropivacaine on pathomorphological changes of the sciatic nerve in DPN rats (**A**) Histopathology changes of sciatic nerve in rats (HE, 40×). (**B**) Histopathology changes in sciatic nerve in rats (Weil’s myelin staining, 40×). (**C**) Observation of sciatic nerves in rats by transmission electron microscope (10k×). (**a**) Nondiabetic control group; (**b**) nondiabetic group A; (**c**) nondiabetic group B; (**d**) diabetic control group; (**e**) diabetic group A; (**f**) diabetic group B.

The pathomorphological changes of the sciatic nerve of rats in each group were determined and are shown in Figure 3A. The lengthwise section of sciatic nerve from a rat from the nondiabetic control group and a rat from the nondiabetic group A showed myelinated nerve fibers that were closely arranged. The myelin tissue was normal, with Schwann cells scattered on the edge of the myelin sheath. In nondiabetic group B, swelling appeared in a few of the nerve fibers and Schwann cells were scattered on the edge of the myelin sheath. The Schwann cells and scabbard cells were slightly reduced in number compared with those of the nondiabetic control group. In the diabetic control group, the nerve fibers were swollen, with only a few normal myelin sheaths. There were fewer scabbard cells, and axons were observed in the myelin sheath. In rats from diabetic group A, some sciatic nerve fibers were swollen, and fewer scabbard cells partly lacked the myelin sheath. In diabetic group B rats, the nerve fibers showed obvious swelling and there was inflammatory cell infiltration. There were few scabbard cells, and some axons showed degradation. There was apparent congestion in the epineurium.

### Effect of ropivacaine on the pathomorphological changes of the sciatic nerve in DPN rats by Weil’s staining

Compared with the nondiabetic control group, the sciatic nerve sheath showed no decrease in scabbard cells, myelinolysis or demyelination in the nondiabetic group A. Samples from the nondiabetic group B showed fewer normal scabbard cells, with myelinolysis or demyelination appearing in part of the myelin sheath. Compared with the nondiabetic control group, rats in the diabetic control group showed some myelinolysis and demyelination with a slight decrease in the number of scabbard cells. The sciatic nerve sheath of the diabetic group A rats showed some partial dissolving and removal of the myelin, with a slight reduction in sheath cells. The samples from diabetic group B showed mostly dissolved myelin sheath and fewer scabbard cells (Figure 3B).

### Effect of ropivacaine on the pathomorphological changes of the sciatic nerve in DPN rats by electron microscopy

In the nondiabetic control group and nondiabetic group A, the sciatic nerve fibers were regularly arranged, with an ordered layer, and clearly visible membranes. The axons were surrounded by myelin, with dense mitochondria, abundant neurons and normal membrane structure. In the nondiabetic group B, loosened myelin sheath was observed, the Schwann cells showed mild edema, and some axons showed atrophy. In the diabetic control group, the myelin sheath was loose. There was typical segmental demyelination, with rare demyelination in patches. The mitochondria of the nerve cells were swollen. In diabetic group A rats, complete separation of the myelin sheath layers was observed in the sciatic nerve. In diabetic group B rats, the sciatic nerves showed swollen myelin sheaths with denatured vacuolar structure. Demyelination and severe axonal atrophy were observed in some sciatic nerves (Figure 3C).

### Effects of ropivacaine on TRPV1 expression in the spinal cord dorsal horn of DPN rats by immunohistochemical assay

**Figure 4:**
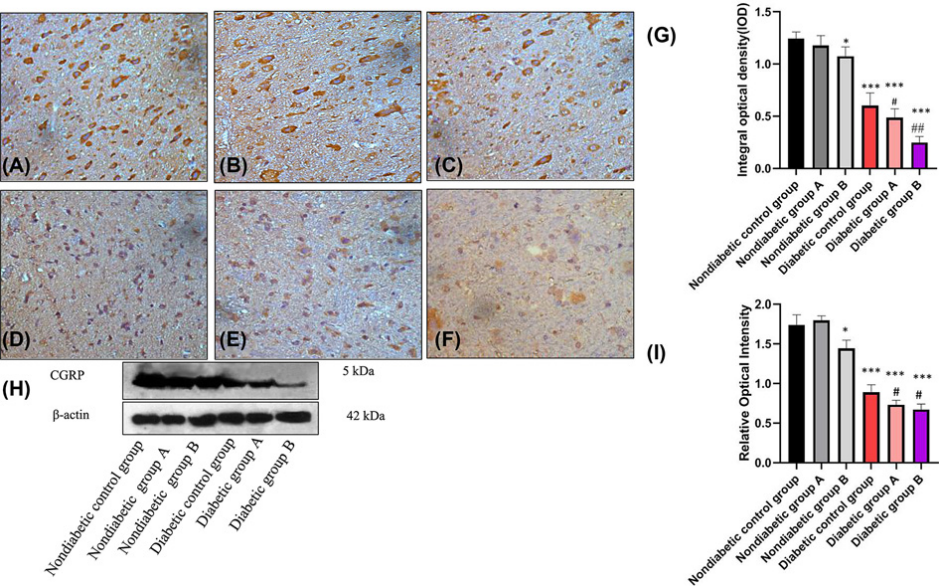
Effects of ropivacaine on the TRPV1-CGRP pathway in the spinal dorsal root ganglion of DPN rats (**A**–**G**) Effect of ropivacaine on the expression of TRPV-1 in spinal dorsal horn in rats (40×). (A) nondiabetic control group; (B) nondiabetic group A; (C) nondiabetic group B; (D) diabetic control group; (E) diabetic group A; (F) diabetic group B; (G) proportion of immuno-positive cells in each group. Values are expressed as mean values ± S.E.M, *n*=8 in each group. Compared with the nondiabetic control group, ***P<0.001; compared with the diabetic control group, ^#^*P*<0.05. (**H,I**) Effect of ropivacaine on the expression of CGRP protein in spinal cord in rats. (H) Western blot images representing the expression of CGRP in each group; (I) comparison of gray value between each group. Values are expressed as mean ± S.E.M. (*n*=8 in each group). Compared with the nondiabetic control group, *P<0.05, ***P<0.001; compared with the diabetic control group, ^#^*P*<0.05,^ ##^*P*<0.01.

With successive injection of ropivacaine, fewer immuno-positive cells were present in rats in nondiabetic group B compared with rats from the nondiabetic control group (*P*<0.05). After establishment of the DNP rat model, there were fewer TRPV-1 protein immuno-positive cells in the diabetic control group and diabetic group A than in the nondiabetic control group rats. Very few TRPV-1 protein immuno-positive cells were seen in diabetic group B samples (*P*<0.001; Figure 4A–G).

### Effects of ropivacaine on CGRP expression in the spinal cord of DPN rats by Western blot assay

Compared with the nondiabetic control group, the nondiabetic group B showed decreased expression of CGRP (*P*<0.05). With successive injection of ropivacaine, the diabetic groups also showed decreased expression compared with the nondiabetic control group (both *P*<0.001). Compared with the diabetic control group, diabetic group B (*P*<0.05) showed a greater decrease in expression of CGRP (Figure 4H,I).

## Discussion

STZ-induced diabetic rodents are widely used to study DPN [20], Our results show that STZ-induced diabetic rats exhibit hyperglycemia, decreased body weight gain and mechanical allodynia after STZ injection at day 56 (8 weeks), supporting the use of STZ-induced diabetic rats as an animal model to study the mechanism of type 1 DPN.

Patients with diabetes may have different complications as the disease progresses, such as retinopathy, foot disease, cardiovascular disease and DPN. Peripheral neuropathy is a common late complication of diabetes, with 60% prevalence [21]. However, the pathogenesis of DPN has not been fully elucidated. Along with hyperglycemia and inositol reduction, nonenzymatic glycosylation, lipid metabolism, oxidative stress-related disorders, microcirculation disorders, hemorheology abnormalities and vasoactive factor abnormalities can affect nerves and the peripheral blood and nutrient levels. Yu et al. [22] from China and England reported the potential neurotoxic effects of ropivacaine in a rodent model of type 1 diabetes mellitus with confirmed DPN. They found that ropivacaine was slightly more toxic to nerves of neuropathic animals when compared with the effects on nerves of control animals. However, the majority of patients undergoing surgery and certainly most neuropathic patients worldwide, are type 2 diabetes mellitus. Cuvillon et al. [2] studied the duration of a sciatic nerve block (0.475% ropivacaine) in type 2 diabetic patients compared with nondiabetic patients, the motor block (16 vs 12 h, *P*<0.01) were higher in the diabetic group. The dose of ropivacaine used (ropivacaine 0.5%, 0.2 ml) seems adequate [23]. There was slight evidence of nerve injury when 0.5% ropivacaine was used. Anecdotal evidence lists several patients who experienced worsening of neurological function after peripheral or neuraxial anesthesia. The extent of pre-existing DPN in these patients ranged from subclinical to severe, and included diabetic neuropathies [24]. Although it is difficult to link the mechanism of neural ischemia-edema produced by local anesthetics (ropivacaine) toxicity and that seen in diabetes mellitus, likely it is the addition of both effects that may cause the emergence of transient or permanent nerve injury in these patients who receive regional anesthesia/analgesia techniques [25]. In the present study, the use of 0.25% ropivacaine did not show significant neurological damage in nondiabetic group, however, the use of 0.75% ropivacaine produced significant neurological damage with or without DPN. CGRP may participate in this process.

CGRP was first identified as an active peptide in 1983 by Rosenfeld et al. [3]. It is a 37-amino acid peptide, with a molecular weight of 3.8 kDa and a biological half-life of 18 min [26]. CGRP is abundant in the endings of spinal dorsal horn primary afferent fibers, spinal ganglion cells, and the spinal nerve dorsal root, and is distributed to the tissues and organs of the cardiovascular system and kidneys through peripheral nerves [27]. Neuropathy and sensory degeneration are common complications of diabetes mellitus [28,29] and reduced levels of CGRP have been reported in diabetes patients [30,31].

CGRP is present in various systems of the human body, has potent physiological activity, and is significant as an indicator for diagnosis and treatment of disease, including peripheral neuropathy [32]. In the CNS, the spine contains the highest levels of CGRP, and the cerebral cortex has very little CGRP. The enhanced expression of CGRP may promote injured peripheral nerve regeneration [33]. After peripheral nerve injury, immature Schwann cells proliferate rapidly, allowing phagocytosis of the myelin sheath and collapsed axons. Macrophages and bands of Schwann cells also participate in the regeneration. In the present study, we observed that 0.75% ropivacaine can inhibit the expression of CGRP in the lumbar spinal cord of nondiabetic or diabetic rats. This effect may directly affect the conduction velocity of the sciatic nerve. The sciatic nerve samples of the diabetic control group showed decreased number of swollen nerve fibers and sheath cells. After the intervention of ropivacaine, especially in diabetic group B, there was more obvious swelling of the sciatic nerve fibers, apparent occlusion of the sciatic cells, and evidence of vacuolization and myelination by electron microscope. These studies indicate that 0.75% ropivacaine is potentially harmful to peripheral nerves, and this phenomenon is more pronounced in diabetic rats. However, it remains unclear if CGRP affects the function of nerve cells or acts in nerve regeneration.

A recent study suggested that CGRP may provide direct protection to neurons, though the mechanism has not been determined [34]. There are several potential explanations. First, CGRP plays a role in maintaining the intracellular ion calcium level. By reducing the permeability of the cellular membrane to calcium, CGRP may protect the brain against hypoxia of cerebral neurons. Second, CGRP may mediate the voltage dependence of calcium channels, causing accelerated release of relevant transmitters [35]. Third, CGRP may up-regulate expression of the Bcl protein and thus protect neurons [36]. Previous work found that CGRP can be activated by the TRPV1 pathway [37].

In 1997, Priestley et al. [38] first cloned and isolated TRPV1, a nonselective cation channel with six transmembrane domains. Activated TRPV1 can promote the calcium-dependent release of transmitters such as substance P and CGRP in peripheral nerve endings [39]. The opening of the TRPV1 ion channel allows entry of positive ions, especially calcium, resulting in an increase in the intracellular level. Vesicles containing CGRP are thus activated and continue to release CGRP to complete transmission [40]. Ren et al. [41] reported that for patients with diabetes complicated by cardiovascular disease, there was decreased expression of TRPV1, causing a decrease in the secretion and release of the SP and CGRP peptides by TRPV1. This was also verified using STZ-induced diabetic rats, suggesting that activation of TRPV1 and the release of CGRP participate in diabetic vascular disease, and circulatory function directly affects peripheral neuropathy progress. Immunohistochemical experiments showed the expression of TRPV1 in the diabetic groups exhibited varying degrees of reduction, compared with the nondiabetic control group. High concentration (0.75%) of ropivacaine most significantly down-regulated TRPV-1 in the spinal dorsal horn. Permpoonputtana et al. [42] studied the RT4-D6P2T Schwann cell line *in vitro* and found that inhibition of CGRP or its receptors may activate the cAMP-PKA/ERK cascade amplification signal system to promote inflammatory factor IL-1β, which plays an important regulatory role in the inflammatory reaction of peripheral nerves. TRPV1 involvement in CGRP release has also been confirmed [43].

In conclusion, the effect of ropivacaine on the motor conduction velocity of the peripheral sciatic nerves may be through decreasing TRPV-1 in the dorsal horn, thereby reducing the release of CGRP in the spinal cord. The reduction in CGRP interferes with the homeostasis of Ca^2+^ in sciatic nerve cells, likely by decreasing the Ca^2+^ permeability of the cell membrane or possibly by affecting the voltage-dependent Ca^2+^ current, thereby inhibiting the release of neurotransmitters and slowing nerve conduction. However, additional evidence is needed to confirm this hypothesis.

## Conclusions

The animal model of experimental DPN was successfully established by intraperitoneal injection of STZ. Ropivacaine significantly inhibited the motor conduction velocity of the sciatic nerve in DPN rats in a concentration-dependent manner. TRPV1 and CGRP are key players in DPN pathology. Ropivacaine may aggravate the blocking of the DPN nerve by affecting the expression of CGRP.

## Abbreviations

CGRP: calcitonin gene-related peptide
CNS: central nervous system
DAB: diaminobenzidine
DM: diabetes mellitus
DPN: diabetic peripheral neuropathy
HE: hematoxylin and eosin
MNCV: motor nerve conduction velocity
PNS: peripheral nervous system
RIPA: radio immunoprecipitation assay
STZ: streptozotocin
TRPV: transient receptor potential vanilloid
